# Life expectancy and risk of death in 6791 communities in England from 2002 to 2019: high-resolution spatiotemporal analysis of civil registration data

**DOI:** 10.1016/S2468-2667(21)00205-X

**Published:** 2021-10-13

**Authors:** Theo Rashid, James E Bennett, Christopher J Paciorek, Yvonne Doyle, Jonathan Pearson-Stuttard, Seth Flaxman, Daniela Fecht, Mireille B Toledano, Guangquan Li, Hima I Daby, Eric Johnson, Bethan Davies, Majid Ezzati

**Affiliations:** a Department of Epidemiology and Biostatistics, School of Public Health, Imperial College London, London, UK; b MRC Centre for Environment and Health, School of Public Health, Imperial College London, London, UK; c UK Small Area Health Statistics Unit, Imperial College London, London, UK; d Abdul Latif Jameel Institute for Disease and Emergency Analytics, Imperial College London, London, UK; e Mohn Centre for Children's Health and Wellbeing, School of Public Health, Imperial College London, London, UK; f Department of Statistics, University of California, Berkeley, CA, USA; g London School of Hygiene & Tropical Medicine, London, UK; h Department of Computer Science, University of Oxford, Oxford, UK; i Department of Mathematics, Physics and Electrical Engineering, Northumbria University, Newcastle-upon-Tyne, UK; j Regional Institute for Population Studies, University of Ghana, Accra, Ghana

## Abstract

**Background:**

High-resolution data for how mortality and longevity have changed in England, UK are scarce. We aimed to estimate trends from 2002 to 2019 in life expectancy and probabilities of death at different ages for all 6791 middle-layer super output areas (MSOAs) in England.

**Methods:**

We performed a high-resolution spatiotemporal analysis of civil registration data from the UK Small Area Health Statistics Unit research database using de-identified data for all deaths in England from 2002 to 2019, with information on age, sex, and MSOA of residence, and population counts by age, sex, and MSOA. We used a Bayesian hierarchical model to obtain estimates of age-specific death rates by sharing information across age groups, MSOAs, and years. We used life table methods to calculate life expectancy at birth and probabilities of death in different ages by sex and MSOA.

**Findings:**

In 2002–06 and 2006–10, all but a few (0–1%) MSOAs had a life expectancy increase for female and male sexes. In 2010–14, female life expectancy decreased in 351 (5·2%) of 6791 MSOAs. By 2014–19, the number of MSOAs with declining life expectancy was 1270 (18·7%) for women and 784 (11·5%) for men. The life expectancy increase from 2002 to 2019 was smaller in MSOAs where life expectancy had been lower in 2002 (mostly northern urban MSOAs), and larger in MSOAs where life expectancy had been higher in 2002 (mostly MSOAs in and around London). As a result of these trends, the gap between the first and 99th percentiles of MSOA life expectancy for women increased from 10·7 years (95% credible interval 10·4–10·9) in 2002 to reach 14·2 years (13·9–14·5) in 2019, and for men increased from 11·5 years (11·3–11·7) in 2002 to 13·6 years (13·4–13·9) in 2019.

**Interpretation:**

In the decade before the COVID-19 pandemic, life expectancy declined in increasing numbers of communities in England. To ensure that this trend does not continue or worsen, there is a need for pro-equity economic and social policies, and greater investment in public health and health care throughout the entire country.

**Funding:**

Wellcome Trust, Imperial College London, Medical Research Council, Health Data Research UK, and National Institutes of Health Research.

## Introduction

Health and health inequalities are receiving unprecedented attention in the UK and other advanced economies for at least two reasons. First, inequalities in health, income, and education have undermined social cohesion and created economic and political uncertainty.^1^, ^2^ Second, the COVID-19 pandemic has rapidly and starkly increased mortality and revealed its inequalities.^3^, ^4^, ^5^ As a result, national and local governments, international agencies, and politicians across the political spectrum have stated the need to address inequalities and drawn up plans in which to do so.

Current data for trends in mortality and longevity in the UK are restricted to medium-sized areas such as local authority districts (median population approximately 140 000),^6^ or to aggregations of communities based on socioeconomic measures such as deciles of deprivation.^7^ High-resolution data are limited to snapshots in time by aggregating data from 5 years to obtain stable estimates,^8^, ^9^ and do not present information on trends. The absence of consistent, high-resolution trend data limits our ability to target policies and interventions for addressing health inequalities, and to measure the effects of such policies including the so-called levelling up policies, which aim to address geographical inequalities in the UK.^10^

We aimed to measure mortality and longevity with high spatial and temporal granularity. We used vital registration data with information on location of residence and applied Bayesian statistical methods to analyse trends and inequalities in life expectancy and risk of death at different ages for 6791 middle-layer super output areas (MSOAs) in England, UK, from 2002 to 2019.

**Research in context**

**Evidence before this study**

We searched PubMed for articles published from database inception up to May 13, 2021, using search terms “life expectancy at birth” AND (“sub-national” OR “small area” OR “local”) AND (“trend” OR “time”) AND (“England” OR “United Kingdom”) for papers that had analysed trends in life expectancy in England's communities, with no language restrictions. We also searched for relevant reports through the websites of the Office for National Statistics and Public Health England. We found articles and reports on trends in life expectancy for local authority districts or for aggregations of communities based on socioeconomic measures such as deciles of deprivation. We also found four reports on snapshots of life expectancy for middle-layer super output areas (MSOAs), which had combined data for 5 years to overcome the issue of small numbers of deaths. None of these had analysed trends over time.

**Added value of this study**

To our knowledge, we present high-resolution data for trends in mortality and longevity for all small-area geographies in England for the first time. By using a Bayesian spatiotemporal model based on patterns of mortality over age, space, and time, we obtained robust yearly estimates of mortality by age group for small geographies, together with the uncertainty in these estimates.

**Implications of all the available evidence**

High-resolution spatiotemporal data reveal that a substantial number of communities in England had a decline in life expectancy in the years preceding the COVID-19 pandemic. The extent of inequalities in life expectancy has increased since the beginning of the millennium. The health policy challenge in England is to not only improve health in communities with poor health but also to avoid a reversal of health gains made in the 20th century.

## Methods

### Study design and data

We performed a high-resolution spatiotemporal analysis of civil registration data in which we extracted de-identified data for all deaths in England from 2002 to 2019 (8 646 878 death records) from the UK Small Area Health Statistics Unit research database. The UK Small Area Health Statistics Unit holds approval from the Health Research Authority Confidentiality Advisory Group under regulation 5 of the health service (Control of Patient Information) regulations 2002 (section 251; reference 20/CAG/0028), and the National Research Ethics Service: London-South East Research Ethics Committee (reference 17/LO/0846).

MSOA of residence was determined using postcode of residence at death registration. We used MSOA boundaries from the 2011 census, which divide England into 6791 MSOAs, with a median population of 7985 (5th–95th percentile 5760–11 917) in 2019. Deaths were stratified into the following age groups: 0, 1–4, 5–9, 10–14, then 5-year age groups up to 80–84, and 85 years and older. We did not use 129 death records (<0·001%) for which sex was not recorded. Mid-year population data by MSOA, age group, year, and sex were obtained from the UK Office for National Statistics.^11^ In 48 (0·001%) age-MSOA-year combinations, the number of deaths exceeded population. Most of these were in people aged 85 years and older. In these cases, the population was set equal to the number of deaths.

We used data for the following measures of socioeconomic deprivation from the English Indices of Deprivation:^12^ income deprivation (referred to as poverty hereafter; the proportion of MSOA population claiming income-related benefits due to being out of work or having low earnings); employment deprivation (referred to as unemployment hereafter; the proportion of the relevant population of the MSOA involuntarily excluded from the labour market due to unemployment, sickness or disability, or caring responsibilities); and education, skills and training deprivation (referred to as low education hereafter; lack of attainment and skills, including education attainment levels, school attendance, and language proficiency indicators), in the MSOA population.

The above measures are the three largest contributors to the Index of Multiple Deprivation, excluding a domain on health that also uses mortality data.^12^ We used data for these measures for 2004, as data for 2002 were not available, and 2019 to analyse how estimated (posterior) life expectancy in 2002 and 2019 was associated with socioeconomic deprivation at the beginning and end of the analysis period. We used the rank of each socioeconomic measure in each year (with values from one to 6791) because their absolute values are not comparable in different years. We did not stratify by ethnicity because data are only available for the 2011 census year. We used classification of MSOAs into rural or urban from the Office for National Statistics.

### Statistical analysis

The number of deaths in each age group, MSOA, and year is small, which means that death rates calculated from observed data have an apparent variability from year to year, or from MSOA to MSOA, which is larger than the true differences in the risk of death. We used a Bayesian hierarchical model to obtain stable estimates of death rates by sharing information across age groups, MSOAs, and years. We conducted all analyses separately by female and male sexes because mortality and trends differ by sex.

In our hierarchical model, death rates for each age group, MSOA, and year were informed by data in that MSOA-age-year unit as well as by those in the adjacent age groups, adjacent years, and nearby MSOAs (ie, those in the same district, followed by other districts in the same region). The extent to which the estimated death rates are influenced by other MSOA-age-year units depend on the number of deaths, with more populous MSOAs and age groups having more influence from their own data than smaller MSOAs and age groups, which are informed by the combination of their own data and data in other units. The model was formulated to take into account how death rates vary in relation to age, time, and geography. Specifically, we allowed each age group to have a different level (ie, intercept) and trend (ie, slope with respect to time) in log-transformed death rate. We specified age group intercepts and slopes with a random-walk structure over age to allow for non-linear age associations. This specification also avoids implausible age patterns of mortality, which could occur if each age group were analysed separately.^13^ We also included MSOA intercepts and slopes, which represent the levels and trends of death rates in each MSOA. The MSOA intercepts and slopes had a hierarchical structure with MSOAs nested in local authority districts, which themselves are nested within regions. We included age-MSOA interaction terms. These terms allow the association of death rates with MSOA to vary by age group (eg, more or less variation across MSOAs in some ages than others) and, equivalently, each MSOA can have a different age pattern of mortality. Finally, because time trends in death rates can be non-linear, we specified time trends of log-transformed death rates using linear terms, as specified above, plus non-linear terms for each age group and MSOA via random walks. Detailed model specification is presented in the appendix (pp 3–5).

In addition to a hierarchical model, we tried a fully spatial Besag, York, and Mollie model so that information is shared both locally (among neighbouring MSOAs) through spatially structured random effects with a conditional autoregressive prior, and globally through spatially unstructured Gaussian random effects. The results of the spatial model were virtually identical to the hierarchical model (correlation coefficient 0·999 for female and male sexes; mean difference 0·03 years for women and 0·009 years for men; mean absolute difference 0·07 years for women and 0·09 years for men for life expectancy estimates from the two approaches). We present results from the hierarchical model for two reasons. First, it allows neighbouring MSOAs that fall in different districts to differ more than those within the same district, reflecting the relevance of district as a unit of resource allocation and policy implementation. Second, the hierarchical model was computationally less demanding with run times about 1·4 times faster than the spatial model.

In 2017, the MSOA in Kensington and Chelsea, London where Grenfell Tower is located had 119 deaths, compared with 48 in 2016 and 51 in 2018; the additional deaths were caused by a fire in a high-rise residential building. This outlier year led to unstable estimates of the long-term trend in life expectancy in this MSOA, and also slightly changed estimates in other MSOAs in the district. To avoid this instability, when applying the statistical model, we replaced the number of deaths for this year with the mean of those in 2016 and 2018 for each age and sex group. When making estimates for 2017, the difference between actual and interpolated deaths was added back to the posterior estimates so that these deaths were counted in the corresponding year.

We fitted the model using the Bayesian model fitting software NIMBLE,^14^, ^15^ and obtained 1000 draws from the posterior distribution of model parameters, which were used to calculate age-specific death rates and life expectancy. Details of model fitting, including the number of chains, length of burn-in, and thinning are provided in the appendix (p 5). We calculated life expectancy at birth, and probability of dying at specific ages by sex and MSOA using life table methods. Life expectancy in a specific year measures the expected length of life if age-specific death rates are the same as those in that year. We used the Kannisto-Thatcher method to expand the terminal age group (≥85 years) of the life table. The reported 95% credible intervals (CrIs) represent the 2·5th to 97·5th percentiles of the posterior distribution of estimated life expectancies. We also report the posterior probability that the estimated change over time in an MSOA represents an increase versus a decrease in life expectancy. Posterior probability represents the inherent uncertainty in life expectancy trends. If the estimated life expectancy is the same in 2002 and 2019 and an increase is statistically indistinguishable from a decrease, there is a 50% posterior probability of an increase and a 50% posterior probability of a decrease. In an MSOA in which the entire posterior distribution of life expectancy in 2019 is greater than in 2002, there is around a 100% posterior probability of an increase, and hence around a 0% probability of a decrease, and vice versa. Posterior probabilities more distant from 50% indicate more certainty. All data management and model fitting were performed using R software, version 3.6.3.

### Role of the funding source

The funders of the study had no role in study design, data collection, data analysis, data interpretation, or writing of the report.

## Results

**Figure 1:**
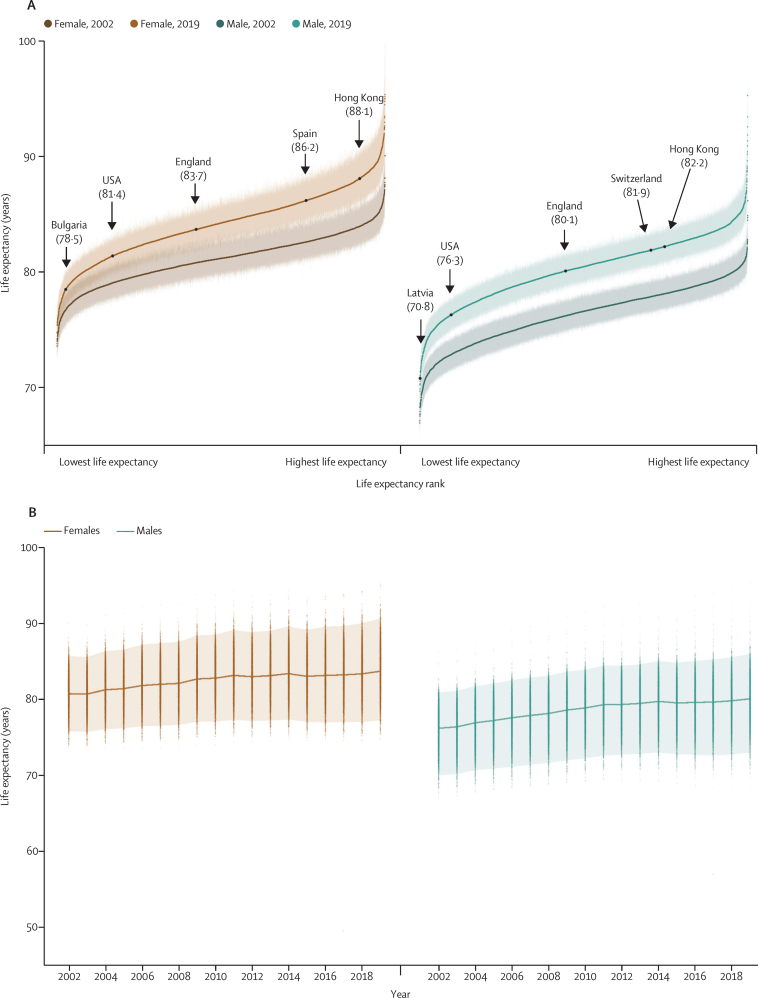
Life expectancy in 6791 MSOAs, 2002–19 (A) Ranked MSOA life expectancies in 2002 and 2019. Each point shows the posterior median life expectancy estimate for each MSOA, forming a curve; error bars are 95% credible intervals. Arrows indicate national life expectancies in England and selected comparator countries with life expectancies within the range of English MSOAs. Hong Kong had the highest global female and male life expectancies. In the EU, Bulgaria had the lowest and Spain had the highest life expectancies for women; Latvia had the lowest and Switzerland had the highest life expectancies for men. Life expectancy for England was calculated from the the UK Small Area Health Statistics Unit research database, and for other countries from World Bank estimates in 2019. (B) Distribution of MSOA life expectancies in each year from 2002 to 2019. Each point shows one MSOA and the upper and lower lines show the first and 99th percentiles of life expectancy. The height of the shaded area is the first to 99th percentile range. The central line shows national life expectancy. MSOA=middle-layer super output area.

**Figure 2:**
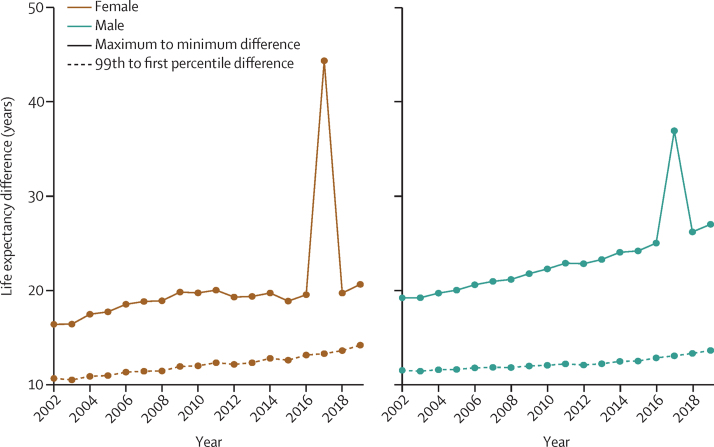
Maximum (highest) to minimum (lowest) and 99th to first percentile differences in life expectancy across 6791 MSOAs, 2002–19 The large difference in 2017 is due to the low life expectancy in the MSOA where the deaths in the Grenfell Tower (Kensington and Chelsea, London) fire took place. MSOA=middle-layer super output area.

**Figure 3:**
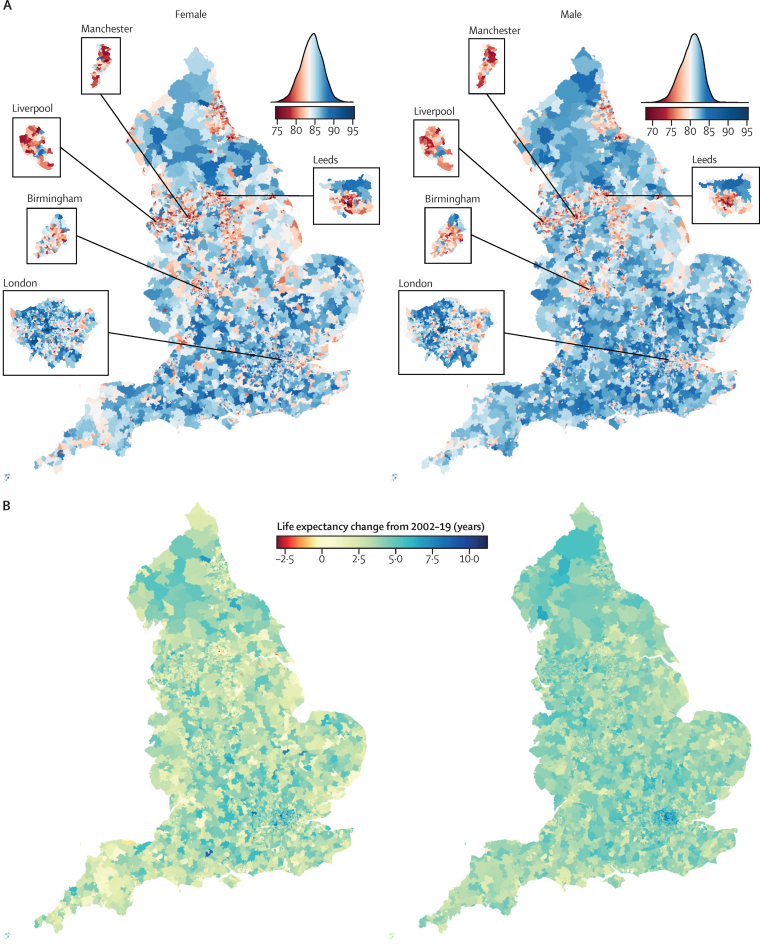

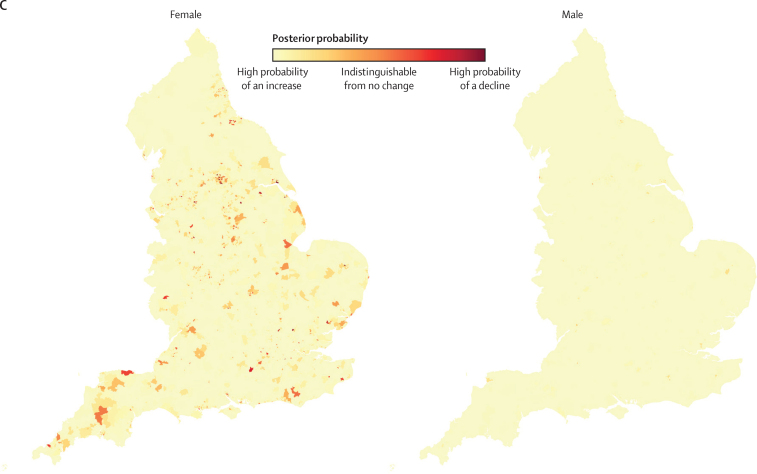
Geography of life expectancy in 6791 MSOAs in England, 2002–19 (A) Map of life expectancy and the distribution of life expectancy in 2019. (B) Change in life expectancy from 2002 to 2019. (C) Posterior probability that the estimated change represents a true increase or decrease in life expectancy from 2002 to 2019. In A, the areas in white have a life expectancy equal to the national life expectancy. In C, posterior probability represents the uncertainty in estimated life expectancy change. MSOA=middle-layer super output area.

In 2019, there was a 20·6 year (95% CrI 17·5–24·2) gap for women between the MSOA with the highest life expectancy (an MSOA in Camden, London; 95·4 years [92·4–98·7]) and the MSOA with the lowest life expectancy (an MSOA in Leeds; 74·7 years [73·4–76·2]; Figure 1, Figure 2, Figure 3). The gap was 27·0 years (23·4–31·1) for men, between an MSOA in Kensington and Chelsea, London (95·3 years [92·1–99·3]) and an MSOA in Blackpool (68·3 years [66·9–69·6]). The difference between the first and 99th percentiles of life expectancy in 2019 was 14·2 years (95% CrI 13·9–14·5) for women and 13·6 years (13·4–13·9) for men. When all MSOAs were ranked on the basis of their life expectancy, the difference between successively ranked MSOAs was particularly large for the approximately 5% of MSOAs with the lowest and highest life expectancy (seen as the sharper decline or rise at the two ends of the ranked life expectancy curve in figure 1A), indicating distinct groups at extreme advantage and disadvantage.

**Figure 4:**
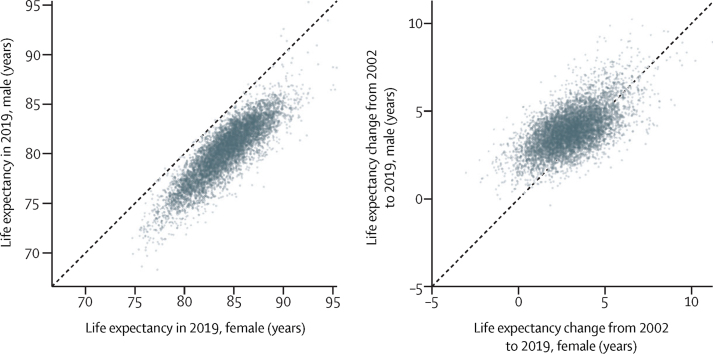
Comparison of female and male life expectancy Life expectancy in 2019 (A) and change from 2002 to 2019 (B).

The 124 (1·8%) of 6791 MSOAs with the lowest female life expectancy and 262 (3·9%) MSOAs with the lowest male life expectancy in 2019 were located in urban areas, particularly in the north, including Blackpool, Leeds, Liverpool, Manchester, and Newcastle. Many of the MSOAs with the highest life expectancy, especially for men, were in London and its neighbouring districts. Female and male life expectancy were correlated across MSOAs with a correlation coefficient of 0·87 (figure 4). Female life expectancy was higher than male life expectancy in all but 15 MSOAs. The female advantage was more than 5 years in 1498 (22·1%) of 6791 MSOAs and 1–5 years in another 5187 (76·4%). By contrast, the mean difference between male and female life expectancies in the 15 MSOAs with higher male life expectancy was 0·72 **ye**ars. Although formal statistical tests for these sex differences cannot be made because female and male analyses were performed separately, in all cases of an apparent male advantage there was substantial overlap between the posterior distributions for the two sexes.

From 2002 to 2019, a decline in life expectancy was more probable than an increase in 124 mostly urban MSOAs of 6791 (1·8% of all MSOAs) for women, with posterior probabilities of greater than 80% that these were true declines in 34 of these. The largest estimated decline of 3·0 years (95% CrI 0·9–5·3; posterior probability of the estimated decline being a true decline 99·6%) occurred in an MSOA in Leeds (figure 3B, C). Elsewhere, median posterior change was positive, ranging from less than 1 year in 408 MSOAs to more than 7 years in 63 MSOAs. Posterior probability of an increase in male life expectancy was more probable than a decrease in all but one MSOA in Blackpool, in which life expectancy changed by −0·4 years (−2·3 to 1·6; posterior probability of being a true decline 64%). For the other MSOAs, the increase ranged from less than 1 year in 31 MSOAs to more than 7 years in 114 MSOAs. The largest increases in female and male life expectancies were seen in some MSOAs in and around London (eg, in the London Borough of Camden). In 5133 (75·6%) MSOAs, male life expectancy increased more than female life expectancy (figure 4), leading to a closing of the life expectancy gap between female and male sexes.

**Figure 5:**
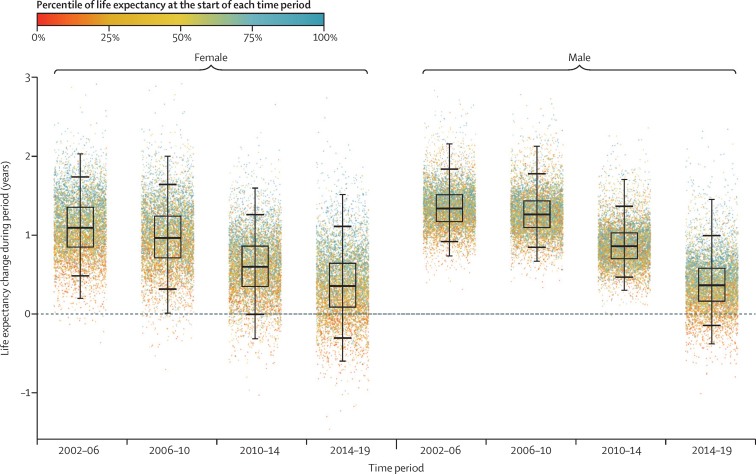
Change in MSOA life expectancy in different time periods, 2002–19 Each point shows the posterior median change in one MSOA. MSOAs are coloured by their life expectancy at the beginning of each period (eg, for 2014–19, they are coloured by life expectancy in 2014). The inner box shows the 25th, 50th, and 75th percentiles, and the outer lines the first, fifth, 95th, and 99th percentiles. MSOA=middle-layer super output area.

The life expectancy increase from 2002 to 2019 was smaller in MSOAs where life expectancy had been lower in 2002, and vice versa, especially for women, which led to a larger life expectancy inequality across MSOAs in 2019 than in 2002 (figure 1A). Specifically, the aforementioned 20·6 year (95% CrI 17·5–24·2) gap for women and 27·0 year (23·4–31·1) gap for men between the lowest and highest MSOA life expectancies in 2019 were larger than those in 2002 by 4·3 years (−1·3 to 9·3) for women and 7·7 years (4·0 to 11·7) for men. Similarly, the gap between the first and 99th percentiles of MSOA life expectancy for women increased from 10·7 years (95% CrI 10·4–10·9) in 2002 to reach 14·2 years (13·9–14·5) in 2019, and for men increased from 11·5 years (11·3–11·7) in 2002 to 13·6 years (13·4–13·9) in 2019. When broken down by time period, the vast majority of MSOAs saw a life expectancy increase in 2002–06 and 2006–10 (figure 5). By contrast, women in 351 (5·2%) MSOAs had a median posterior change in life expectancy in 2010–14 that was negative. By 2014–19, the number of MSOAs with a negative median posterior change had risen to 1270 (18·7%) for women, with men in 784 (11·5%) MSOAs also showing a decline. These MSOAs tended to be places in which life expectancy was already low.

**Figure 6:**
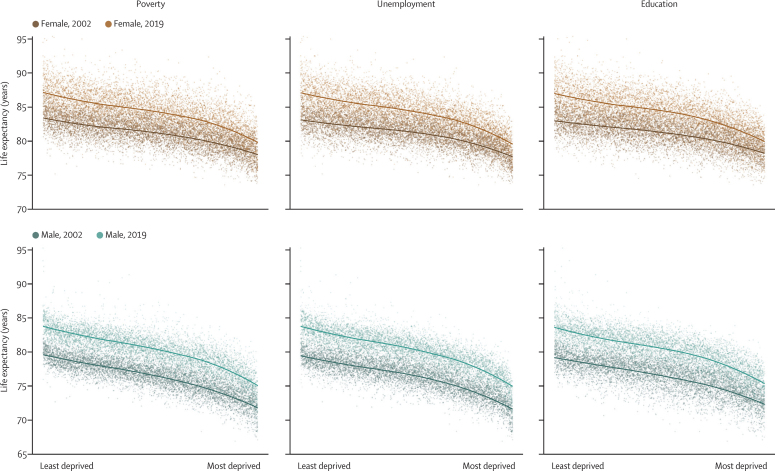
MSOA life expectancy in relation to measures of socioeconomic deprivation in the MSOA in 2002 and 2019 The socioeconomic measures are poverty, unemployment, and education, as defined in the Methods. The lines show the smooth relationship fitted with locally estimated scatterplot smoothing for each year. MSOA=middle-layer super output area.

Life expectancy at birth was inversely associated with the extent of unemployment, poverty, and low education in MSOA in 2002 and 2019 (figure 6). There was substantial variation in life expectancy across MSOAs at any level of poverty or unemployment seen in the vertical spread of points in figure 6. From 2002 to 2019, there were, on average, smaller gains in life expectancy in the MSOAs with the highest levels of unemployment, poverty, and low education than in those in the lowest levels, especially for women.

Similar to life expectancy, there were large inequalities in the probability of surviving from birth to 80 years, which ranged from 42% to 87% in women and 27% to 85% in men across MSOAs in 2019. These large survival inequalities were present at every stage of the life-course including childhood and early adolescence (0–15 years), young adulthood (15–30 years), working ages (30–70 years), and older ages (70–80 years; appendix pp 15–16). Specifically, the probability of dying at different stages of the life-course in the 99th percentile of MSOAs was between 2·6 and 3·1 times that of the first percentile for female and male sexes in 2019. From 2002 to 2019, the relative inequality across MSOAs (ie, ratio of the 99th to the first percentile) in the probabilities of dying increased at every stage of the life course; the absolute inequality (ie, difference between the 99th and first percentiles) decreased slightly in all combinations except for working age women (30–70 years). Within childhood and adolescence, there were particularly large inequalities in infant mortality (0 to <12 months), with a ratio of the 99th to the first percentile of MSOAs being 3·2 for female and male sexes in 2019. Infant mortality increased from 2014 to 2019 in 1378 (20·3%) MSOAs for girls and 888 (13·0%) for boys, many of which experienced a decline in life expectancy.

## Discussion

Our high-resolution analysis over space and time shows that life expectancy has not only ceased to increase but has declined in many communities in England since 2010. The decline has accelerated since 2014, affecting the female population of 18·7% of MSOAs and the male population of 11·5% of MSOAs. In 1·8% of MSOAs, women have had a long-term decline in life expectancy over two decades. MSOAs that have gained the least in longevity since 2002 were those that started with the lowest life expectancy, located in northern urban areas with high levels of poverty and unemployment, and with relatively low education. Conversely, those MSOAs with higher life expectancies in 2002 had some of the largest gains. As a result, England has seen widening inequalities in longevity, with the life expectancy gap surpassing 20 years for women and 27 years for men.

The main strength of our study is the presentation of high-resolution data for mortality and longevity across England over a period of substantial change in economic, health, and social care policy. By applying a hierarchical model based on patterns of mortality over age, space, and time, we obtained robust yearly estimates of mortality and life expectancy, together with the uncertainty in these estimates, for small areas. By contrast, studies that had not used a coherent model produced unstable (ie, very large uncertainty) or implausible life expectancy estimates in some MSOAs, despite having aggregated deaths over 5 years, nor could they analyse trends at the MSOA level.^8^, ^9^ Comparison of estimates at MSOA and district level shows that the estimated MSOA life expectancy range was about 1·8 times the district-level range for women and 2·0 times the district-level range for men in 2019.

A limitation of our work is that we did not analyse underlying causes of death, which should be the subject of further research to reveal the diseases and injuries driving the differences in mortality trends. We did not break down age beyond 85 years, which might mask some differences in old-age mortality and survival patterns. Although MSOAs have small populations and are designed to have some socioeconomic homogeneity, there are inevitable variations in socioeconomic status and health within them. To understand life expectancy inequalities in relation to individual socioeconomic characteristics requires linking health and other data such as census records, education, and taxes, as done in countries like New Zealand and Sweden. Furthermore, the people who live in each MSOA can change due to both within-country and international migration. Migration estimates for geographical units with consistent boundaries are only available at the district level for 2012–17.^16^ Regression of the change in life expectancy from 2012 to 2017 in each MSOA (excluding the MSOA containing Grenfell Tower, which was an extreme outlier in 2017) against the mean rate of population inflow and outflow in the district containing the MSOA in those years explained only 8% of the variation in life expectancy change for women and 16% for men at the national level. Studies in both the UK^17^ and USA^18^ have also shown that migration is not sufficient to explain the trends in health and health inequalities, and that these trends are largely due to real changes in population health. Even if rising inequalities are partly due to health-selective migration, this phenomenon has social and economic origins that should be addressed through employment opportunities, affordable housing, high-quality education, and health care. Population and mortality statistics in the UK are generated independently from one another. As a result, we encountered a situation of having more deaths than population in a small percentage (0·001%) of age-MSOA-year combinations, a phenomenon that was more common in those aged 85 years and older. This finding might be due to errors in population estimates in years between censuses or because some people (eg, those living in long-term care facilities such as care homes), are counted in one MSOA for the population statistic but have their death registered in another. Furthermore, care home residents might have relocated from other MSOAs, with different socioeconomic characteristics from that in which the care home is located. This factor could attenuate the association between socioeconomic variables and life expectancy. The extent of this underestimation is modest; however, because a large part of life expectancy variation is due to deaths at earlier ages, when people are less likely to live and die in care homes.^7^ Finally, statistical models that remove (unwanted) variability due to small populations by sharing information across spatial units, can also attenuate true variation, a phenomenon known as shrinkage. In our analysis, the extent of shrinkage is likely to be modest (appendix pp 17–18) because we used data for multiple age groups and years in each MSOA. Nonetheless, the true extent of inequality in life expectancy across MSOAs is likely to be larger than estimated here. Similarly, the random walk prior and random effects distributions used for sharing information across age groups could attenuate variability in some age groups while inflating it in others. For instance, when we analysed death rates separately by the age groups shown in the appendix (pp 15–16), the estimated inequality in the risk of dying became slightly larger in working ages (30–70 years), and slightly smaller in children and adolescents (0–15 years), young adults (15–30 years), and older ages (70–80 years).

Our life expectancy estimates in specific years are similar to the snapshots presented by the Office for National Statistics^8^ and Public Health England,^9^ with correlation coefficients of 0·92–0·95 and mean differences of −0·004 to 0·19 years. However, these reports could not analyse trends because data were aggregated over 5 years (2009–13, 2013–17, or 2015–19). In terms of trends, national studies have recorded a slowdown in mortality improvement since 2010,^19^ and those that grouped small-area units into deciles of deprivation have detected a decline in female life expectancy in the one or two most deprived deciles.^2^, ^7^ By analysing trends at the MSOA level, we could identify the communities in which longevity is declining and show that the decline, which began around 2010 in women in some MSOAs, has spread and accelerated since 2014.

Over the period of this analysis, from 2002 to 2019, national life expectancy increased in high-income countries in Australasia, Europe, and North America. Female life expectancy has stagnated or declined in various intervals since 2010 in the UK (84% of the UK population in 2019 lived in England) and in some other high-income countries including France, Germany, Italy, and the USA;^20^, ^21^ the UK and USA have had some of the poorest performances in terms of the duration or extent of slowdown or reversal in longevity gain.^21^, ^22^ The comparative performance of high-income countries' longevity trends has been attributed to differences in risk factors such as smoking, health care, and social inequalities.^21^, ^22^ Since the 2008 economic crisis, there has also been attention on how cuts to social and health services in austerity budgets might have affected health in different countries.^21^, ^22^, ^23^, ^24^

To our knowledge, nationwide trend data for small-area life expectancy are available only in the USA. The declining life expectancy in numerous English MSOAs since 2014, especially those that already had a low life expectancy, resembles a trend spanning nearly three decades in the USA in two ways.^18^, ^25^, ^26^ First, in both countries, there is substantial variation in life expectancy at any level of poverty, which might be due to geographical variations in health behaviours, the public health programmes that influence these behaviours or otherwise prevent disease, and health services.^26^, ^27^, ^28^, ^29^, ^30^ The second similarity in small-area life expectancy trends is that the decline in life expectancy was more widespread in women than in men.^18^ Historically, women and men had similar life expectancies in high-income nations before a rise in traffic injuries and diseases associated with specific occupations and health behaviours such as smoking and alcohol use created a male mortality disadvantage in the 20th century.^31^ The closing of female and male life expectancy in the late 20th century and early 21st century in many high-income nations^32^ is partly due to the dynamics of smoking, which peaked later in women than in men, and affects causes of death such as respiratory diseases and lung cancer that have stagnated or even increased in women in deprived communities.^7^, ^21^ However, it is rare for the convergence of female and male life expectancies to occur in the form of female life expectancy decline,^18^, ^33^ which might be due to a combination of the worsening economic, psychosocial (eg, poverty, stress, and domestic violence), and behavioural (smoking and alcohol use) determinants of mortality in English women.

There has been much attention on how poverty and the underfunding of public health have been associated with the large and unequal mortality toll from the COVID-19 pandemic in England and the USA.^3^, ^4^, ^5^ Our results show that numerous communities in England had begun to have a decline in longevity before the pandemic, mirroring an earlier trend in the USA. In both countries, the decline in life expectancy was associated with the economic trends of unemployment and insecure and low-wage employment following late 20th century deindustrialisation. In England, these economic trends led to a larger loss of jobs in the north than in London and the southeast,^1^, ^4^, ^10^, ^34^, ^35^, ^36^ where improvements in state education have given students, including from poorer areas, skills for jobs in a changing economy.^37^, ^38^ These long-term changes were followed by a reduction in social support and welfare payments and in funding to the local governments during the austerity period, which increased poverty, including in-work poverty,^4^, ^39^, ^40^, ^41^, ^42^, ^43^, ^44^ such that by 2018–19, one in five people in the UK lived in poverty.^45^ These cuts also had larger effects in the north than in London and southern parts of the country and worsened the effects of loss of secure employment.^10^, ^36^, ^41^, ^46^, ^47^ Poverty and reduced funding to services increase mortality through health behaviours such as smoking and alcohol use, poor nutrition and living environment, psychosocial pathways, and lower provision or use of preventive and curative health care.

Although health-care spending in the UK has been less affected by austerity than other sectors, the annual 1·3% increase since 2010 is about a third of the long-term average, and insufficient to keep up with the increasing demand of an ageing population.^48^ The imbalance between funding and demand has led to longer waiting times for primary and specialist care, with the greatest effects in deprived areas.^49^ The real-term cuts in public health spending have also been larger in the north and northeast of England,^50^, ^51^ where life expectancy lags. Smoking cessation and health checks, which affect diseases with substantial contribution to mortality inequalities,^7^ had larger than average funding cuts.^52^

To limit and reverse the falling life expectancy, there is an urgent need for pro-equity economic and social policies, and greater investment in public health and health care especially in communities with low life expectancy. However, the UK's post-Covid Build Back Better agenda does not explicitly address equity. The complementary levelling up funding plans to address geographical inequalities by investing in infrastructure, particularly in the so-called left-behind districts.^10^, ^53^ However, the fund's budget is small relative to its American counterpart, and its prospectus has limited direct focus on child poverty, public health, or high-skilled education. As a result, place-based improvement in northern cities remains limited to local action facilitated by devolution in cities such as Manchester, and community resilience, wellbeing, and regeneration initiatives. These are positive steps but might be insufficient without additional resources for education, employment, and health. Rather, to reverse the decline in longevity in a sizeable segment of England's communities requires making health and health equity a key outcome of any policy and equity-enhancing investment and action in education, secure employment, public health, and health and social care, such as those attempted in the English health inequalities strategy in the 2000s.^54^ Regular reporting of life expectancy with high granularity is essential to identify places in need of intervention and to measure the effects of policies.

## Data sharing

The Small Area Health Statistics Unit does not have permission to release data to third parties except in the form of non-disclosive statistical tables or conclusions suitable for publication. Individual mortality data can be requested through the Office for National Statistics. Mid-year population estimates, urban-rural classifications for each MSOA, and the English Indices of Deprivation are available online. The code for the model and detailed results and visualisation are also available online.

## Declaration of interests

ME reports a charitable grant from the AstraZeneca Young Health Programme, and personal fees from Prudential, outside the submitted work. JP-S is vice-chair of the Royal Society for Public Health and a partner at Lane Clark & Peacock, and reports personal fees from Novo Nordisk, all outside the submitted work. YD is a member of the advisory group for the King's Fund. All other authors declare no competing interests.
