# Supplementary material for: Life expectancy and risk of death in 6791 communities in England from 2002 to 2019: high-resolution spatiotemporal analysis of civil registration data
Source: Lancet Public Health. 2021 Oct 13;6(11):e805–16. doi: 10.1016/S2468-2667(21)00205-X (PMC8554392; doi:10.1016/S2468-2667(21)00205-X)
Supplement: Supplementary appendix [file mmc1.pdf]

THE LANCET
Public Health

## Supplementary appendix

This appendix formed part of the original submission and has been peer reviewed. We post it as supplied by the authors.

Supplement to: Rashid T, Bennett JE, Paciorek CJ, et al. Life expectancy and risk of death in 6791 communities in England from 2002 to 2019: high-resolution spatiotemporal analysis of civil registration data. *Lancet Public Health* 2021; published online Oct 12. http://dx.doi.org/10.1016/S2468-2667(21)00205-X.

**Table of contents**

**Appendix Text 1**: Measures of socioeconomic deprivation for Middle-layer Super Output Areas ........ 2

**Appendix Text 2:** Specification of the Bayesian statistical model ........ 3

**Appendix Figure 1:** Examples of post burn-in and thinning trace plots of death rates. ........ 6

**Appendix Figure 2:** Posterior parameter estimates of the age intercepts together with 95% credible intervals. ........ 8

**Appendix Figure 3:** Posterior parameter estimates of the age time slopes together with 95% credible intervals. ........ 10

**Appendix Figure 4:** Posterior parameter estimates of the age specific nonlinear trend together with 95% credible interval. ........ 12

**Appendix Figure 5:** Probability of dying in specific ages in 6,791 middle-layer super output areas (MSOA) in England in 2002 and 2019. ........ 15

**Appendix Figure 6:** Range of life expectancy between the top and bottom percentiles in 2002 and 2019 estimated using the Bayesian model and the data. ........ 17

**Appendix Table 1:** Specification of the Bayesian statistical model. ........ 19

**Appendix Table 2:** Posterior medians for standard deviations with 95% credible intervals.. 20

**References** ........ 21

**Appendix Text 1**: Measures of socioeconomic deprivation for Middle-layer Super Output Areas

As specified in the main paper, data for the poverty, unemployment and low education indicators were taken from the English Indices of Deprivation.[1] The data are presented at the Lower-layer Super Output Area (LSOA) level – one level smaller in the nested hierarchy of census geographies than the Middle-layer Super Output Area (MSOA) level used in the analysis.[2] Each deprivation domain, including those used here (income deprivation; employment deprivation; education, skills and training deprivation), has a score calculated from administrative data by the Ministry for Housing, Communities & Local Government. However, the definition of the indicators can change over time. Further, the indicator used for measuring education, skills and training deprivation (low education) is not directly interpretable because it combines multiple concepts cannot be simply expressed as a proportion of the population. Therefore, we used ranking so that comparisons can be made not only across MSOAs in a single year, but also across the two years shown in Figure 4 of the main paper.

The 2004 data on deprivation domains were reported for LSOA boundaries from the 2001 census. We mapped these data to the 2011 census LSOA boundaries, which was the reporting unit for the 2019 data, as follows: First, for deprivation, we assigned the 2001 LSOA score to all postcodes contained within it. We then overlayed the 2011 LSOA boundaries, and averaged the score for all constituent postcodes of each LSOA, to obtain the corresponding score for each 2011 LSOA. The MSOA-level scores were created by taking the population-weighted average of scores for all constituent LSOAs, as has been done previously for local authority districts.[3] These were then ranked to obtain the MSOA ranking.

**Appendix Text 2:** Specification of the Bayesian statistical model

As described in the main paper, we used a Bayesian hierarchical model to obtain robust estimates of death rates by age group, MSOA (spatial unit) and year, which were then used to calculate life expectancy. The model was run separately for each sex. The model was formulated to incorporate important features of death rates in relation to age, space and time. Deaths were divided into 19 age groups: 0, 1-4, 5-9, 10-14, …, 80-84 and ≥85 years. The number of deaths in age group $a$ $(= 1, \ldots, 19)$, MSOA $s$ $(= 1, \ldots, 6791)$ and year $t$ $(= 1, \ldots, 18)$ follows a negative binomial distribution

$$Deaths_{ast} \sim \text{Negative Binomial}(p_{ast}, r)\,.$$

The parameter $p_{ast}$ is

$$p_{ast} = \frac{r}{r + m_{ast} \cdot Population_{ast}}\,,$$

where $r(\geq 0)$ is the overdispersion parameter, which accounts for extra variability not captured by other components in the model, and $m_{ast}$ is the death rate. The negative binomial likelihood can be thought of as a generalisation of a Poisson likelihood, which allows for overdispersion, with larger values of $r$ indicating more similarity to a Poisson distribution.

Log-transformed death rates were modelled as a function of time, age group and MSOA. The model contains terms to capture the overall level and rate of change of mortality, as well as age-specific and MSOA-specific terms to allow deviations from these terms. Specifically, log-transformed death rates are modelled as

$$\log(m_{ast}) = \alpha_0 + \beta_0 t + \alpha_{1s} + \beta_{1s} t + \alpha_{2a} + \beta_{2a} t + \xi_{as} + \nu_{st} + \gamma_{at}\,,$$

where $\alpha_0$ is the overall intercept across all age groups and MSOAs. $\beta_0$ quantifies the overall trend (over time) across all age groups and MSOAs. $\alpha_{1s}$ and $\beta_{1s}$ measure deviation from the overall intercept and trend terms, respectively, for each MSOA. $\alpha_{2a}$ and $\beta_{2a}$ measure deviation from the global level and trend, respectively, for each age group. We used first-order random walk priors on $\alpha_{2a}$ and $\beta_{2a}$ so that they vary smoothly over adjacent age groups, with the form

$A_a \sim \mathcal{N}(A_{a-1}, \sigma_A^2)$ for both age-specific terms $\alpha_{2a}$ and $\beta_{2a}$. We constrained $\alpha_{21} = 0$ and $\beta_{21} = 0$ so each random walk was identifiable and centred on the corresponding overall term.

$\xi_{as}$ is an age group-MSOA interaction term, which quantifies MSOA-specific deviations from the overall age group structure given by $\alpha_{2a}$. This allows different MSOAs to have different age-specific mortality patterns, and each age group's death rate to have a different spatial pattern. This interaction term was modelled as $\mathcal{N}(0, \sigma_\xi^2)$.

$\nu_{st}$ and $\gamma_{at}$ are first-order random walks over time that allow MSOA- and age group-specific non-linearity in the time trends. For each MSOA and age group, they were modelled via similar priors to those above with $\nu_{s1} = \gamma_{a1} = 0$ so that the terms were identifiable.

Appendix Table 1 shows all model parameters, their priors and dimensions.

*Spatial structures*

For the main analysis, the MSOA intercepts and slopes, $\alpha_{1s}$ and $\beta_{1s}$, were modelled as nested hierarchical random effects, with MSOAs nested in districts, which were, in turn, nested in regions. The regional terms are centred on zero to allow the spatial effects to be identifiable.

For comparison, we also modelled the spatial effects using a Besag, York and Mollie (BYM) model.[4] The BYM setup models the spatial effects as the sum of spatially-structured random effects with conditional autoregressive (CAR) priors, allowing information to be shared locally between neighbouring MSOAs, and spatially-unstructured (independent and identically distributed, IID) random effects, allowing information to be shared amongst all MSOAs. The CAR component of the model requires all spatial units to have neighbours. Thus, the MSOAs containing the Isle of Wight, Hayling Island, the Isles of Scilly and Canvey Island were each joined to the nearest mainland MSOA based on road or ferry connections.

*Hyperpriors*

As in earlier analyses, weakly informative priors were used so that inference on the parameters was driven by the data.[5,6] All variance parameters of the random effects had $\sigma \sim \mathcal{U}(0,2)$ priors. For the global intercept and slope, we used $\mathcal{N}(0, \sigma^2 = 100000)$. We used these diffuse priors for the global intercept and slope as there is ample information in the data to estimate both parameters. Other diffuse priors, such as a uniform distribution defined on a wide interval, for example from -1000 to 1000, would yield near identical estimates. The overdispersion parameter $r$ had the prior $\mathcal{U}(0,50)$.

*Implementation*

Inference was performed using Markov chain Monte Carlo in NIMBLE.[7,8] Where possible, centred parameterisations were used in the model coding in order to reduce autocorrelation in the chains. We monitored convergence using trace plots and the R-hat diagnostic,[9] and thinned post burn-in samples to reduce memory and storage use. For females we ran four chains for 150,000 iterations, discarding the first 50,000 and thinning the remainder by 400 to obtain 1,000 post-burn-in draws from the posterior distribution of model parameters. For males, due to slower mixing, we ran eight chains for 150,000 iterations, discarding the first 100,000 and thinning the remainder by 400. Since, there are 2,322,522 death rates for each sex it is not possible to show all trace plots. Trace plots of death rates for 6 randomly selected age-space-time combinations are shown for each of males and females in Appendix Figure 1.

*Posterior parameter estimates*

Posterior estimates for the major components of the model are shown in Appendix Figures 2, 3 and 4. Note that these estimates are conditional on all other model parameters and cannot be interpreted in isolation. Posterior summaries of the standard deviations are listed in Appendix Table 2.

**Appendix Figure 1:** Examples of post burn-in and thinning trace plots of death rates. For each of the randomly selected age-space-time combinations there were 4 chains for women and 8 chains for men.

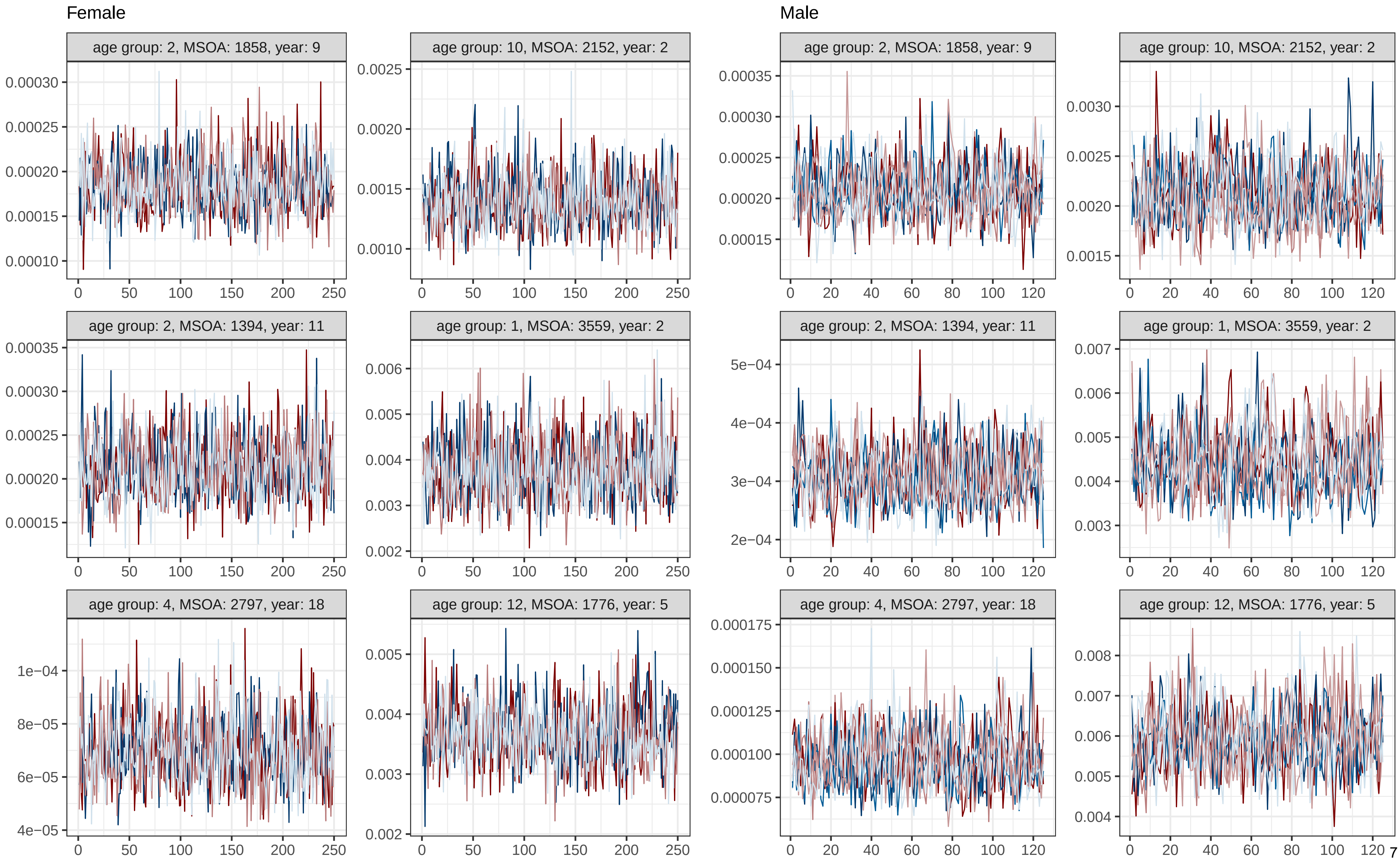
Female
Male
age group: 2, MSOA: 1858, year: 9
age group: 10, MSOA: 2152, year: 2
age group: 2, MSOA: 1394, year: 11
age group: 1, MSOA: 3559, year: 2
age group: 4, MSOA: 2797, year: 18
age group: 12, MSOA: 1776, year: 5

**Appendix Figure 2:** Posterior parameter estimates of the age intercepts $\alpha_{2a}$ together with 50% (blue) and 95% (red) credible intervals.

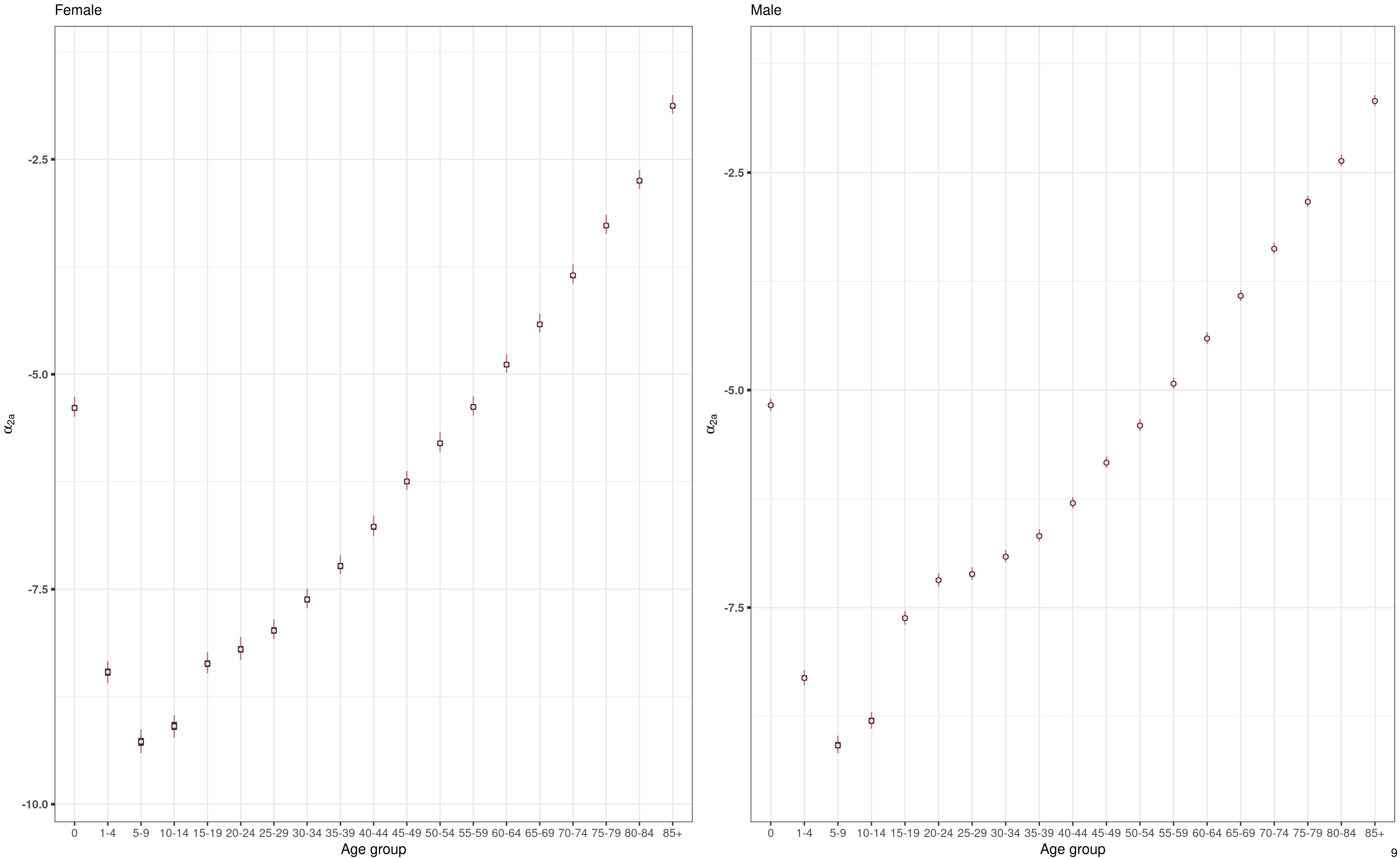
Female
Male
-2.5
-5.0
-7.5
-10.0
$\alpha_{2a}$
0
1-4
5-9
10-14
15-19
20-24
25-29
30-34
35-39
40-44
45-49
50-54
55-59
60-64
65-69
70-74
75-79
80-84
85+
Age group

**Appendix Figure 3:** Posterior parameter estimates of the age time slopes $\beta_{2a}$ together with 50% (blue) and 95% (red) credible intervals.

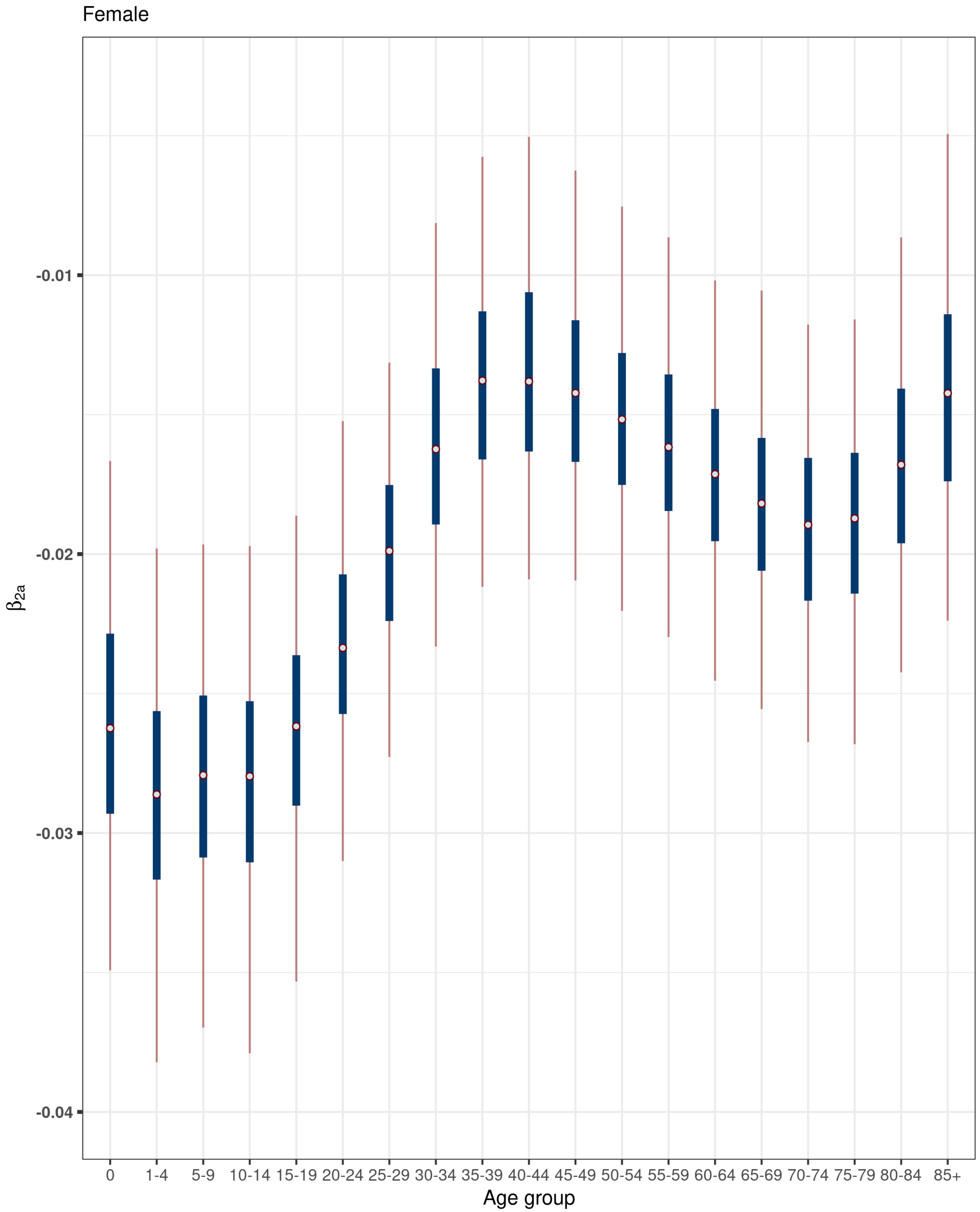
Female
-0.01
-0.02
-0.03
-0.04
β2a
0
1-4
5-9
10-14
15-19
20-24
25-29
30-34
35-39
40-44
45-49
50-54
55-59
60-64
65-69
70-74
75-79
80-84
85+
Age group

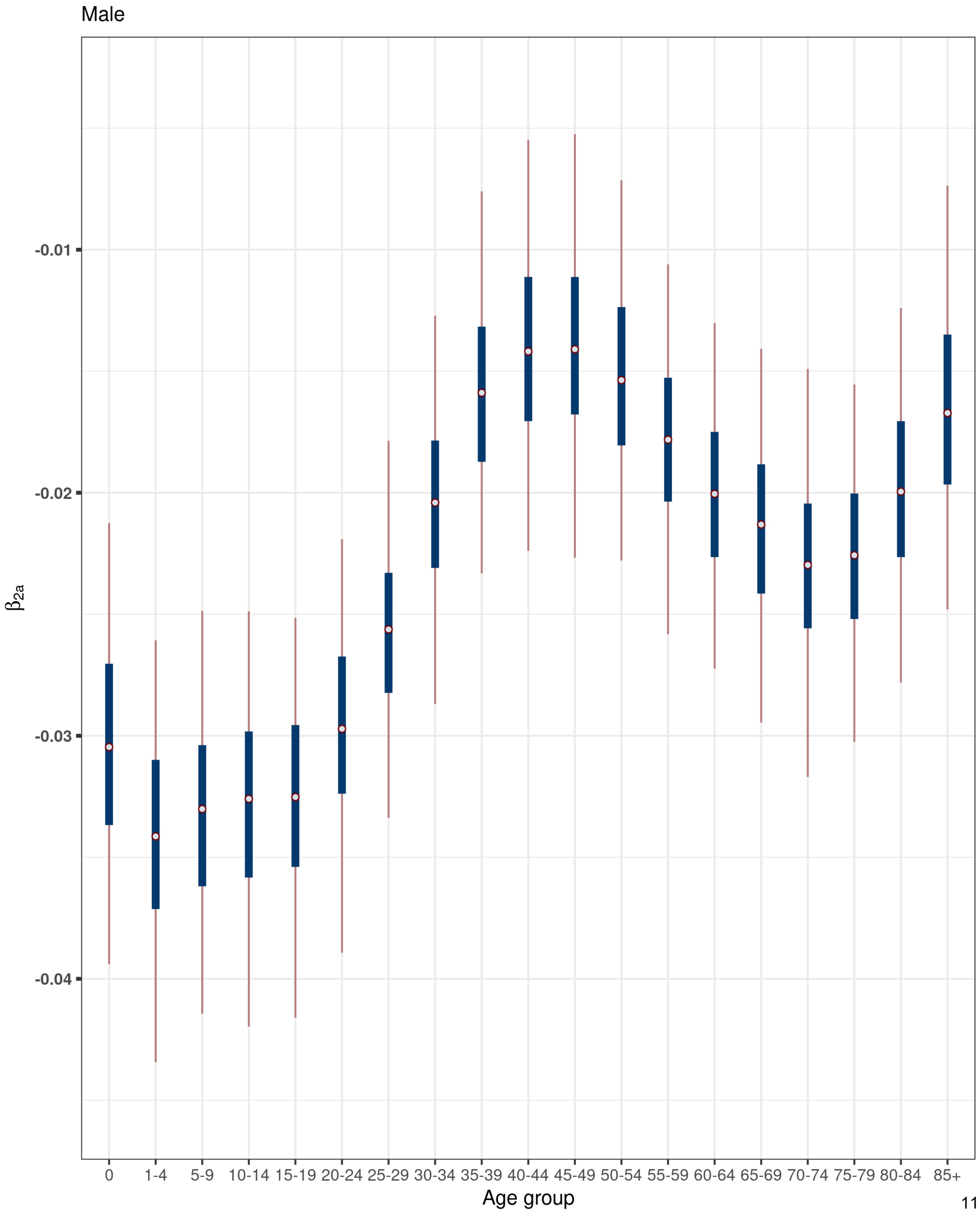
Male
-0.01
-0.02
-0.03
-0.04
β2a
0
1-4
5-9
10-14
15-19
20-24
25-29
30-34
35-39
40-44
45-49
50-54
55-59
60-64
65-69
70-74
75-79
80-84
85+
Age group

**Appendix Figure 4:** Posterior parameter estimates of the age specific nonlinear trend $\gamma_{at}$ together with 50% (blue) and 95% (red) credible interval for females (A) and males (B). Note that due to the way in which the model is coded, this term includes the linear effects $\beta_{2a}$ and hence represents the total of age specific time terms.

A (Female)

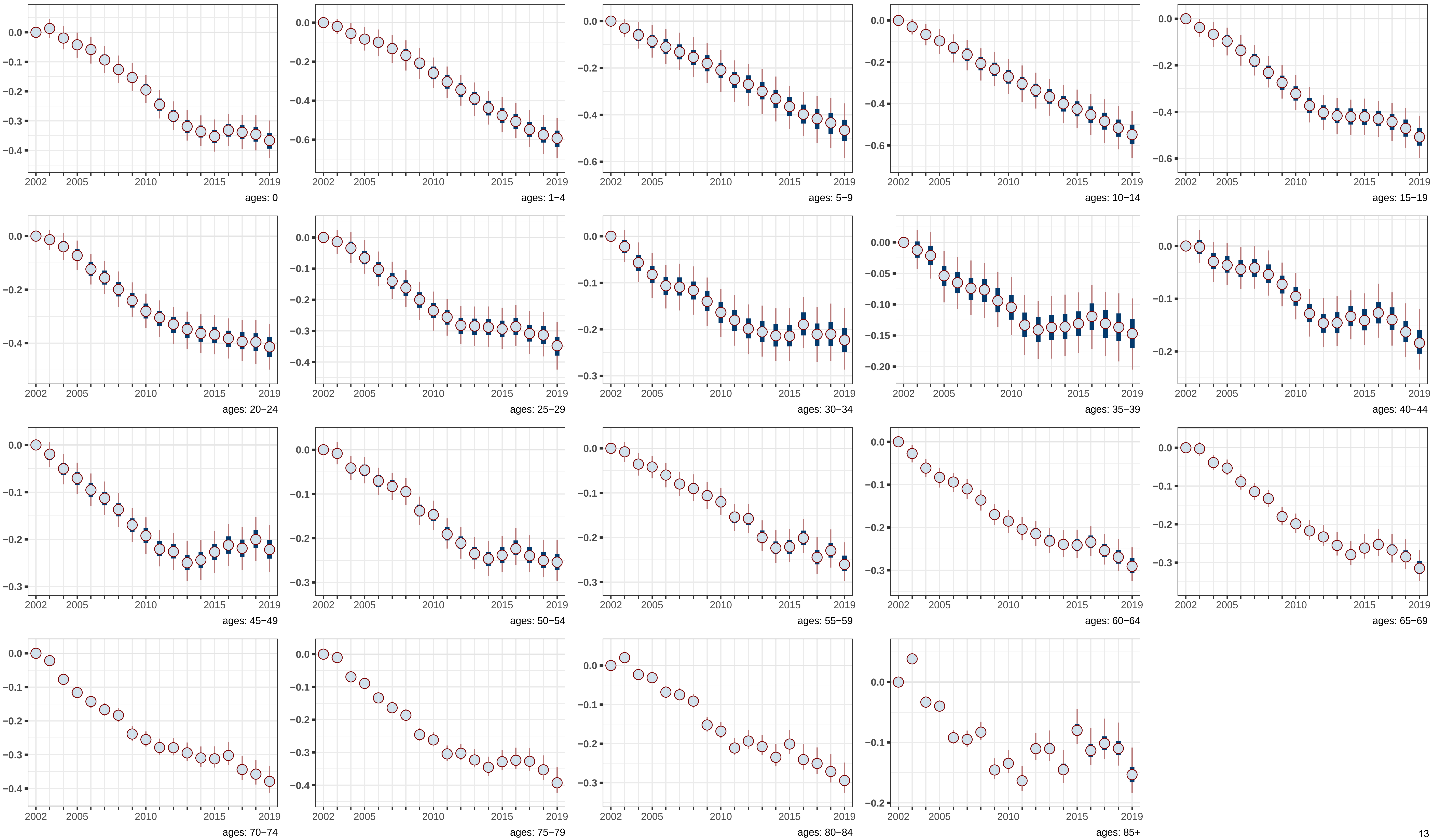
ages: 0
ages: 1−4
ages: 5−9
ages: 10−14
ages: 15−19
ages: 20−24
ages: 25−29
ages: 30−34
ages: 35−39
ages: 40−44
ages: 45−49
ages: 50−54
ages: 55−59
ages: 60−64
ages: 65−69
ages: 70−74
ages: 75−79
ages: 80−84
ages: 85+
2002
2005
2010
2015
2019

B (Male)

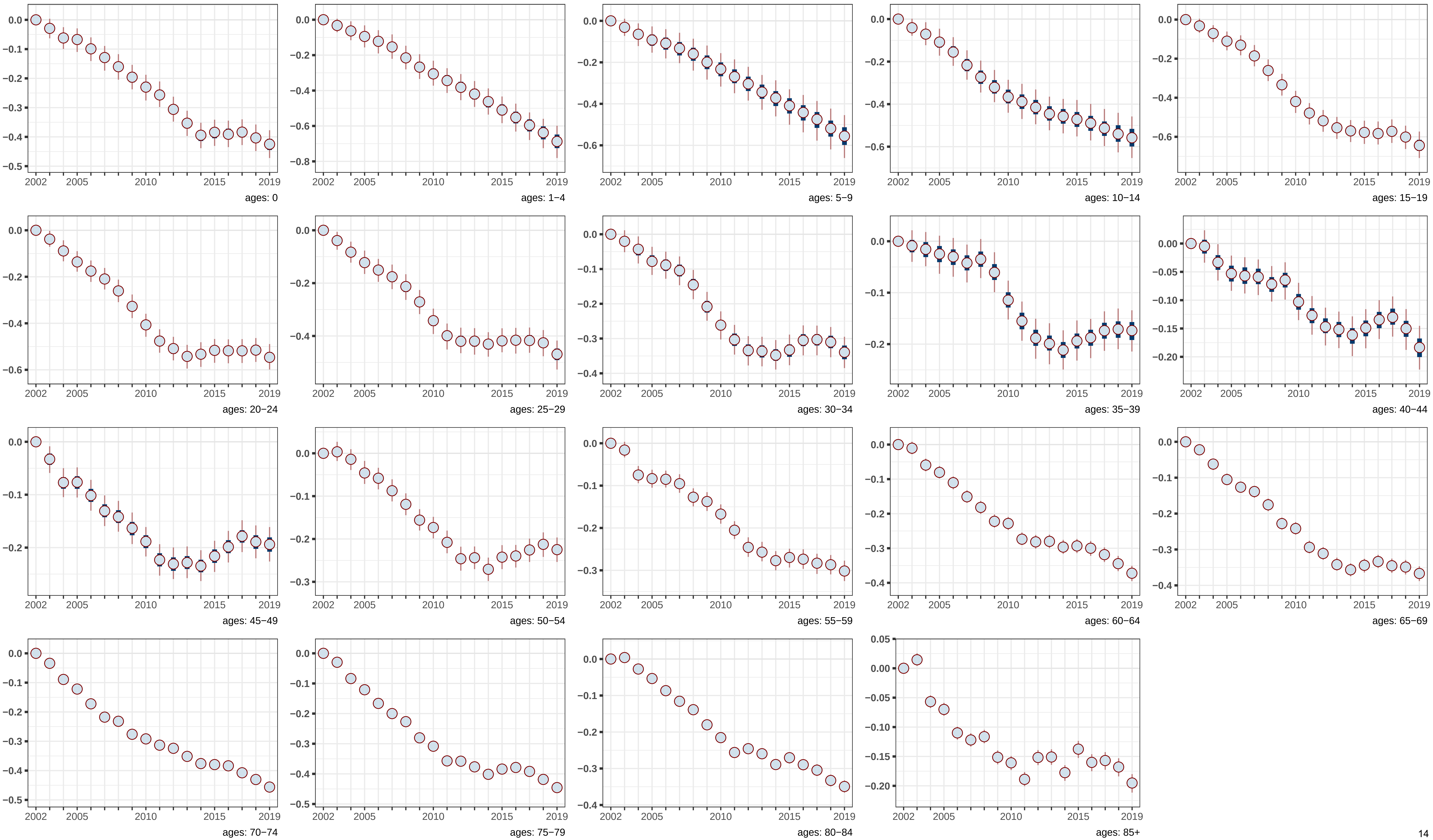
ages: 0
ages: 1−4
ages: 5−9
ages: 10−14
ages: 15−19
ages: 20−24
ages: 25−29
ages: 30−34
ages: 35−39
ages: 40−44
ages: 45−49
ages: 50−54
ages: 55−59
ages: 60−64
ages: 65−69
ages: 70−74
ages: 75−79
ages: 80−84
ages: 85+

**Appendix Figure 5:** Probability of dying in specific ages in 6,791 middle-layer super output areas (MSOA) in England in 2002 and 2019. Each point shows one MSOA. The vertical axis uses a log scale so that the large differences in survival across ages can be seen.

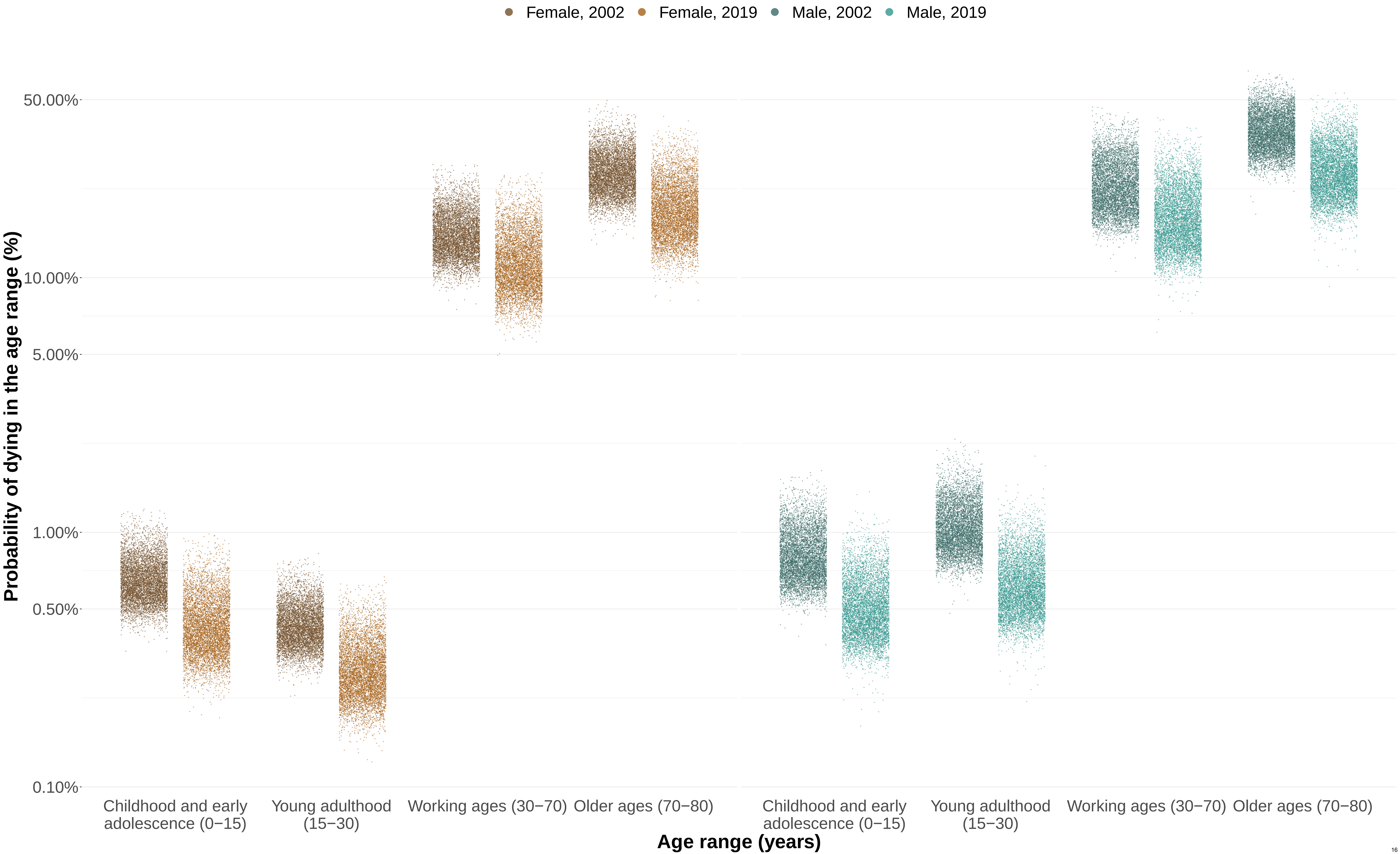

Female, 2002
Female, 2019
Male, 2002
Male, 2019
Probability of dying in the age range (%)
50.00%
10.00%
5.00%
1.00%
0.50%
0.10%
Childhood and early adolescence (0−15)
Young adulthood (15−30)
Working ages (30−70)
Older ages (70−80)
Childhood and early adolescence (0−15)
Young adulthood (15−30)
Working ages (30−70)
Older ages (70−80)
Age range (years)

**Appendix Figure 6:** Range of life expectancy between the top and bottom percentiles in 2002 and 2019 estimated using the Bayesian model and the data.

The top and bottom percentiles in 2002 and 2009 were defined based on the poverty ranking in the years 2004 and 2019, respectively, and each consists of 67 or 68 MSOAs. In both sets of estimates – from the Bayesian model and the data – deaths and population were aggregated across all these MSOAs. The comparison shows that the extent of shrinkage due to smoothing in the Bayesian models is small.

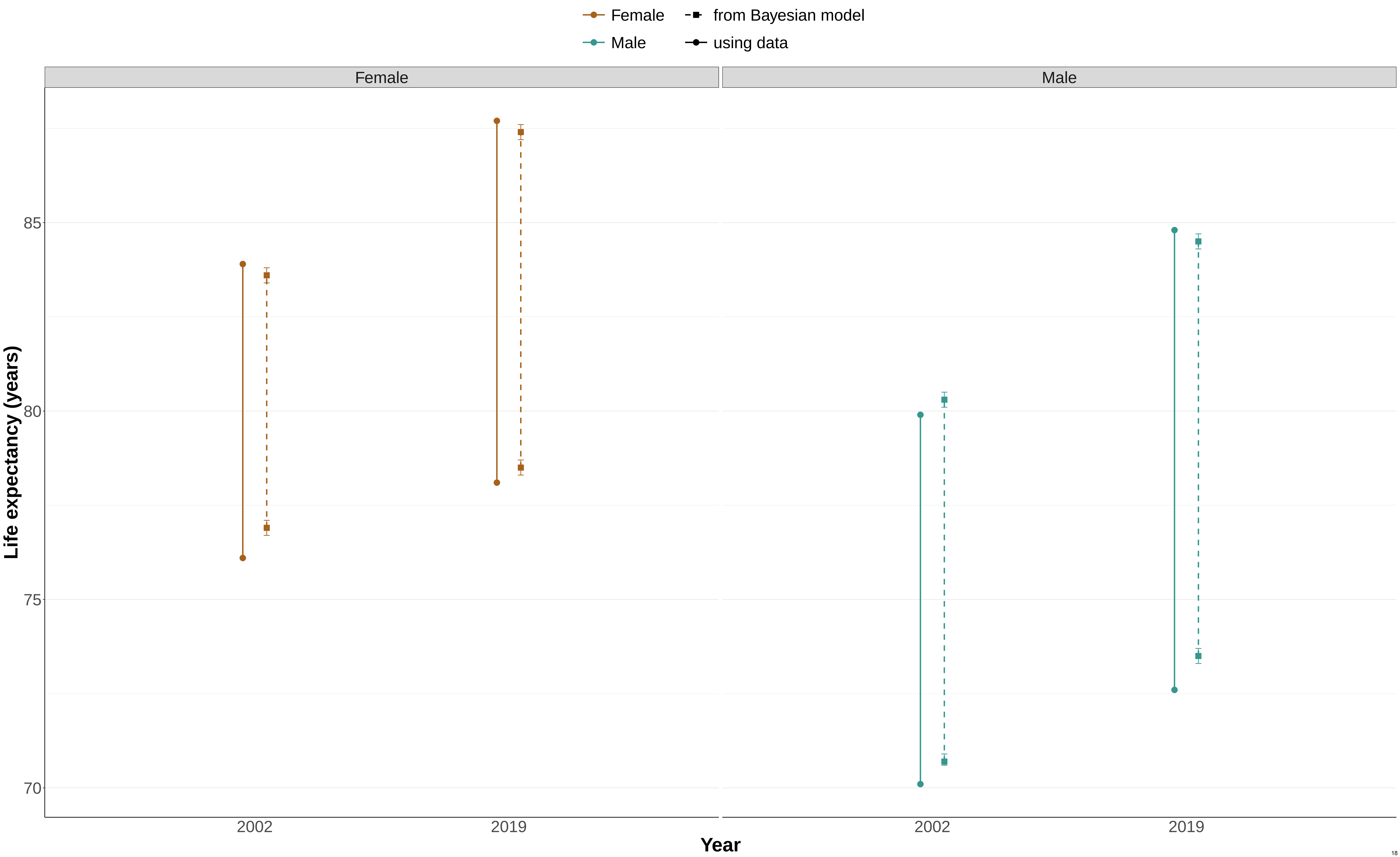

Female
Male
from Bayesian model
using data
Female
Male
Life expectancy (years)
85
80
75
70
2002
2019
2002
2019
Year

**Appendix Table 1:** Specification of the Bayesian statistical model. Subscripts are as follows: s – MSOA; d – district; r – region; a – age group; t – year.

| Parameter name | Symbol | Prior | Dimension |
|---|---|---|---|
| Overall intercept | $\alpha_0$ | $\mathcal{N}(0, \sigma^2 = 100000)$ | 1 |
| Overall slope | $\beta_0$ | $\mathcal{N}(0, \sigma^2 = 100000)$ | 1 |
| Regional intercept | $\alpha_{1r}$ | $\mathcal{N}\left(0, \sigma_{\alpha_{1r}}{}^2\right)$ | 9 |
| Regional intercept standard deviation | $\sigma_{\alpha_{1r}}$ | $\mathcal{U}(0,2)$ | 1 |
| District intercept | $\alpha_{1d}$ | $\mathcal{N}\left(0, \sigma_{\alpha_{1d}}{}^2\right)$ | 314 |
| District intercept standard deviation | $\sigma_{\alpha_{1d}}$ | $\mathcal{U}(0,2)$ | 1 |
| MSOA intercept | $\alpha_{1s}$ | $\mathcal{N}\left(0, \sigma_{\alpha_{1s}}{}^2\right)$ | 6,791 |
| MSOA intercept standard deviation | $\sigma_{\alpha_{1s}}$ | $\mathcal{U}(0,2)$ | 1 |
| Regional slope | $\beta_{1r}$ | $\mathcal{N}\left(0, \sigma_{\beta_{1r}}{}^2\right)$ | 9 |
| Regional slope standard deviation | $\sigma_{\beta_{1r}}$ | $\mathcal{U}(0,2)$ | 1 |
| District slope | $\beta_{1d}$ | $\mathcal{N}\left(0, \sigma_{\beta_{1d}}{}^2\right)$ | 314 |
| District slope standard deviation | $\sigma_{\beta_{1d}}$ | $\mathcal{U}(0,2)$ | 1 |
| MSOA slope | $\beta_{1s}$ | $\mathcal{N}\left(0, \sigma_{\beta_{1s}}{}^2\right)$ | 6,791 |
| MSOA slope standard deviation | $\sigma_{\beta_{1s}}$ | $\mathcal{U}(0,2)$ | 1 |
| Age group intercept | $\alpha_{2a}$ | $\mathcal{N}\left(\alpha_{2,a-1}, \sigma_{\alpha_{2a}}{}^2\right)$ | 18 |
| Age group intercept standard deviation | $\sigma_{\alpha_{2a}}$ | $\mathcal{U}(0,2)$ | 1 |
| Age group slope | $\beta_{2a}$ | $\mathcal{N}\left(\beta_{2,a-1}, \sigma_{\beta_{2a}}{}^2\right)$ | 18 |
| Age group slope standard deviation | $\sigma_{\beta_{2a}}$ | $\mathcal{U}(0,2)$ | 1 |
| Age group MSOA interaction | $\xi_{as}$ | $\mathcal{N}\left(0, \sigma_{\xi}^2\right)$ | 19 x 6,791 |
| Age group MSOA interaction standard deviation | $\sigma_{\xi}$ | $\mathcal{U}(0,2)$ | 1 |
| MSOA random walk over time | $\nu_{st}$ | $\mathcal{N}\left(\nu_{s,t-1}, \sigma_{\nu}^2\right)$ | 6,791 x 17 |
| MSOA random walk over time standard deviation | $\sigma_{\nu}$ | $\mathcal{U}(0,2)$ | 1 |
| Age group random walk over time | $\gamma_{at}$ | $\mathcal{N}\left(\gamma_{a,t-1}, \sigma_{\gamma}^2\right)$ | 19 x 17 |
| Age group random walk over time standard deviation | $\sigma_{\gamma}$ | $\mathcal{U}(0,2)$ | 1 |
| Overdispersion parameter | $r$ | $\mathcal{U}(0,50)$ | 1 |

**Appendix Table 2:** Posterior medians for standard deviations with 95% credible intervals. Subscripts are as follows: s – MSOA; d – district; r – region; a – age group.

| Parameter name | Symbol | Female | Male |
|---|---|---|---|
| Regional intercept standard deviation | $\sigma_{\alpha_{1r}}$ | 0.10 (0.06-0.19) | 0.10 (0.06-0.17) |
| District intercept standard deviation | $\sigma_{\alpha_{1d}}$ | 0.082 (0.076-0.090) | 0.10 (0.09-0.11) |
| MSOA intercept standard deviation | $\sigma_{\alpha_{1s}}$ | 0.17 (0.16-0.17) | 0.19 (0.18-0.19) |
| Regional slope standard deviation | $\sigma_{\beta_{1r}}$ | 0.0034 (0.0022-0.0057) | 0.0030 (0.0020-0.0047) |
| District slope standard deviation | $\sigma_{\beta_{1d}}$ | 0.0032 (0.0028-0.0036) | 0.0028 (0.0025-0.0031) |
| MSOA slope standard deviation | $\sigma_{\beta_{1s}}$ | 0.0064 (0.0059-0.0070) | 0.0063 (0.0056-0.0066) |
| Age group intercept standard deviation | $\sigma_{\alpha_{2a}}$ | 0.92 (0.71-1.28) | 0.95 (0.74-1.32) |
| Age group slope standard deviation | $\sigma_{\beta_{2a}}$ | 0.0040 (0.0017-0.0073) | 0.0049 (0.0025-0.0085) |
| Age group MSOA interaction standard deviation | $\sigma_{\xi}$ | 0.15 (0.15-0.16) | 0.15 (0.15-0.15) |
| MSOA random walk over time standard deviation | $\sigma_{\nu}$ | 0.024 (0.022-0.026) | 0.0095 (0.0043-0.0142) |
| Age group random walk over time standard deviation | $\sigma_{\gamma}$ | 0.025 (0.022-0.028) | 0.026 (0.023-0.029) |

## References

1 Ministry of Housing Communities & Local Government. English Indices of Deprivation 2019. 2019; published online Sept 26. https://www.gov.uk/government/statistics/english-indices-of-deprivation-2019 (accessed Feb 15, 2021).

2 Office for National Statistics. Census geography. 2021. https://www.ons.gov.uk/methodology/geography/ukgeographies/censusgeography (accessed March 31, 2019).

3 Ministry of Housing Communities & Local Government. The English Indices of Deprivation 2019: Technical report. 2019 https://www.gov.uk/government/publications/english-indices-of-deprivation-2019-technical-report.

4 Besag J, York J, Mollié A. Bayesian image restoration, with two applications in spatial statistics. *Ann Inst Stat Math* 1991; **43**: 1–20.

5 Kontis V, Bennett JE, Mathers CD, Li G, Foreman K, Ezzati M. Future life expectancy in 35 industrialised countries: projections with a Bayesian model ensemble. *Lancet* 2017; **389**: 1323–35.

6 Gelman A, Carlin J, Stern H, Dunson D, Vehtari A, Rubin D. Bayesian Data Analysis, 3rd edn. Chapman and Hall/CRC, 2013.

7 de Valpine P, Turek D, Paciorek CJ, Anderson-Bergman C, Lang DT, Bodik R. Programming with Models: Writing Statistical Algorithms for General Model Structures with NIMBLE. *J Comput Graph Stat* 2017; **26**: 403–13.

8 de Valpine P, Paciorek CJ, Turek D, *et al.* NIMBLE: MCMC, Particle Filtering, and Programmable Hierarchical Modeling v0.10.1. 2020. DOI:10.5281/zenodo.1211190.

9 Vehtari A, Gelman A, Simpson D, Carpenter B, Bürkner PC. Rank-normalization, folding, and localization: An improved R-hat for assessing convergence of MCMC. *arXiv* 2019; : 1–28.
